# The variations in the East Asian summer monsoon over the past 3 kyrs and the controlling factors

**DOI:** 10.1038/s41598-019-41359-y

**Published:** 2019-03-22

**Authors:** Katsura Yamada, Kazuma Kohara, Minoru Ikehara, Koji Seto

**Affiliations:** 1 Department of Geology, Faculty of Science, Shinshu University, 3-1-1 Asahi, Matsumoto, 3908621 Japan; 2 Center for Advanced Marine Core Research, Kochi University, B200 Monobe, Nankoku, 7838502 Japan; 3 Estuary Research Center, Shimane University, 1060 Nishikawatsu, Matsue, 6908504 Japan

## Abstract

The mechanisms driving the variations in the centennial-scale East Asian summer monsoon (EASM) remain unclear. Here, we use the δ^18^O records from adult ostracode shells to reconstruct the EASM variations over the last 3 kyrs in southwestern Japan. A common variation with a 200 yr periodicity among the Asian monsoonal regions was recognized between BC 800 and BC 100. Since then, neither a correlation between the EASM variation and solar activity or a common EASM variation through EASM regions has been identified. The evidence reveals that solar activity dominantly affected the centennial-scale EASM variations throughout Asian monsoonal regions until BC 100. Furthermore, factors other than solar activity that varied and differed in specific regions controlled the EASM intensity due to decreasing summer solar insolation in the Northern Hemisphere after BC 100. These relations indicate that the dominant factor that affects the EASM variations shifts according to the solar insolation intensity.

## Introduction

Climates in Asia are strongly controlled by monsoons that are linked to the global climate^1,2^. The East Asian monsoon, which is one of the largest monsoon systems, covers the regions including China, the Korean Peninsula and Japan^3^. The East Asian monsoon is composed of southeastern winds in summer and northwestern winds in winter. The summer monsoon transports a large volume of water vapour to the continents, providing important water resources. High-resolution speleothem records from caves have clarified the variations in the East Asian summer monsoon (EASM) and their relations to other climate and sun patterns. In particular, new records from various regions over the last two decades have improved the understanding of monsoon mechanisms and the driving forces during the Quaternary^4,5^. Numerous EASM records, particularly those from stalagmites in Chinese caves, have indicated that both the orbital-scale and millennial-scale EASM variations are mainly due to solar insolation^4–9^. In addition to sun activity, CO_2_ concentrations^10^, the El Niño–Southern Oscillation (ENSO)^11^, and climate in the high latitudes of the Northern Hemisphere^4,12^ have been proposed to be strongly linked to the intensity of the Asian summer monsoon. However, due to the small number of centennial-scale EASM records compared to the number of orbital- and millennial-scale records, coherent centennial-scale EASM variations and the relationships between the centennial-scale EASM variations and solar activity remain vague.

Regardless of the significance and usefulness of the δ^18^O records in stalagmites, their inconsistencies between nearby caves have been recognized^7,13^. Furthermore, there are controversies that the δ^18^O records in stalagmites from southern China reflect the isotopic compositions of water vapour from the upstream source region and not the EASM intensity in southern China^12,14–17^. Thus, the EASM records derived from other materials are required.

**Figure 1:**
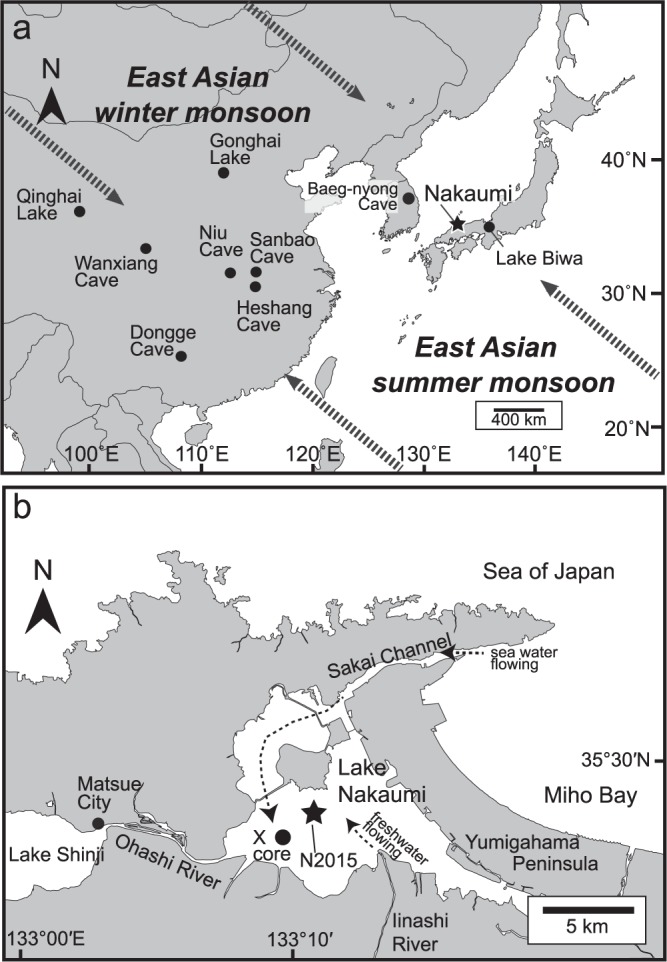
Map showing East Asia and Lake Nakaumi. (**a**) The location of Lake Nakaumi and the regions where the EASM records were recognized. The dotted arrows show the window directions. (**b**) The locations of the N2015 (star) and X (filled circle) cores. The dotted lines indicate the directions of freshwater and seawater inflows.

Ostracoda is a microcrustacea that inhabits aquatic environments throughout the world. The calcareous shells of Ostracoda are well preserved in the bottom sediments of freshwater lakes, brackish lakes and marine environments^18^. The δ^18^O of the adult shell of a brackish species *Bicornucythere bisanensis* has been used as a tool to reconstruct the past summer precipitation induced by the EASM in Lake Nakaumi, southwest Japan^19^ (Fig. 1). According to the EASM records based on this method, centennial-scale EASM variations with periodicities of 300–500 yrs and no relationship with solar activity during the last 1.8 kyrs were defined. This result disagreed with the previous observations that the EASM was strongly linked to solar activities, as found in orbital- and millennial-scale EASM variations. In the present study, we reconstructed the centennial-scale EASM intensity over the last 3 kyrs to reveal the common EASM intensity and its relation to solar activity.

## Results and Discussion

### Regional effect on ostracode δ^18^O values

**Figure 2:**
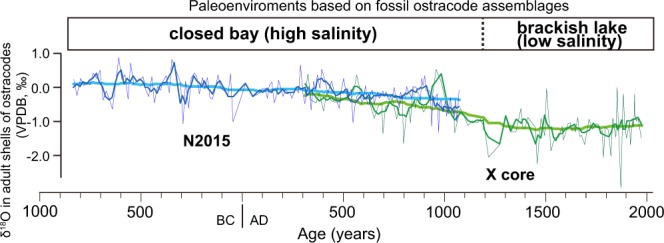
The δ^18^O records from ostracode shells of *Bicornucythere bisanensis*. The data from the N2015 core (blue) were represented by adding the records from the X core (green)^19^. The coloured thick, thin and extra-thin lines suggest 500 yr and 50 yr running mean values and measurement values, respectively. Environmental changes were derived from fossil ostracode assemblages^18^.

The δ^18^O values in the N2015 core from Lake Nakaumi fluctuated between 1.0 and −1.25 (Fig. 2, Supplementary Table S1). The interval over which the δ^18^O records were obtained from the N2015 core overlapped to with the interval for the X core between AD 300 and 1100^19^. The 500 yr running mean values of the ostracode δ^18^O from the N2015 core were similar to those in the X core during the interval; moreover, a common gradually decreasing trend was also found. Overall, the long-term δ^18^O values gradually declined beginning in BC 900. This trend corresponds to a decrease in salinity induced by ostracode assemblage changes^18^, suggesting that the long-term trend in the δ^18^O records of ostracode shells was caused by a regional environmental shift accompanied by a gradual decrease in salinity (Fig. 2).

**Figure 3:**
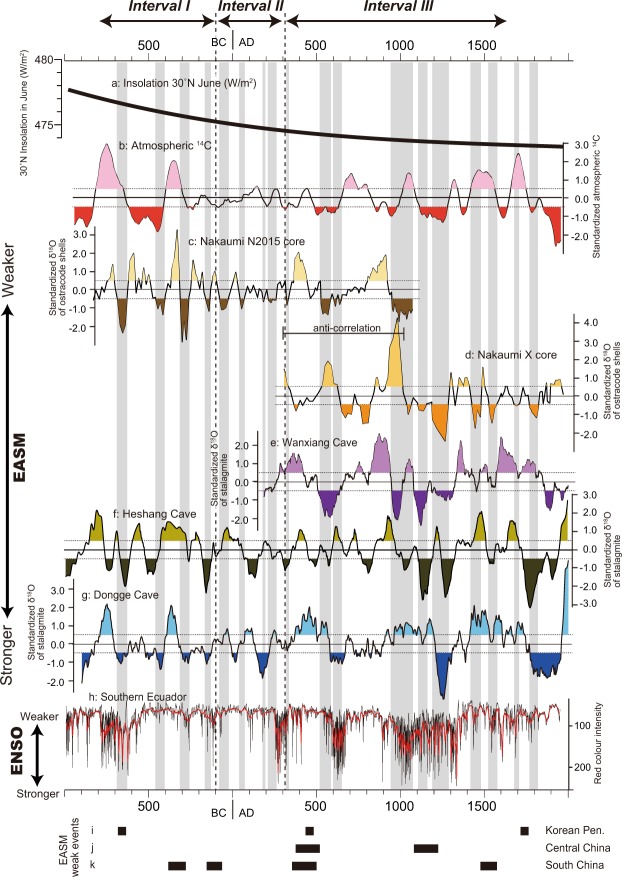
Comparisons of EASM records in the monsoonal region, sun activity and climatic records. (**a**) Insolation at 30°N in June^49^. (**b**) Standardized cosmogenic atmospheric ^14^C^23^. (**c**) Standardized δ^18^O records in ostracode shells of *Bicornucythere bisanensis* from the N2015 core in Lake Nakaumi, southwest Japan. (**d**) Standardized δ^18^O in ostracode shells of *B*. *bisanensis* from the X core in Lake Nakaumi, southwest Japan^19^. (**e**) Standardized δ^18^O records from stalagmites from Wanxiang Cave, central China^20^. (**f**) Standardized δ^18^O records from stalagmites from Heshang Cave, central China^13^. (**g**) Standardized δ^18^O records from stalagmites from Dongge Cave, south China^4^. (**h**) Red colour intensity from the lake in southern Ecuador^36^. (**i**–**k**) weak EASM events identified in the millennial-scale EASM records from Baeg-nyong Cave, Korean Peninsula^25^, Tibetan Plateau, central China^5^, and Dongge Cave, south China^7^. Vertical grey bars indicate enhanced EASM periods from the records in Lake Nakaumi.

The standardized δ^18^O values between the two cores taken from Lake Nakaumi displayed anti-correlation between AD 300 and AD 1000 (Fig. 3). During this period, the reconstructed EASM intensity in the N2015 core showed a positive correlation with that in the δ^18^O records from stalagmites in Wanxiang Cave, China^20^, whereas there was a negative correlation with the records from the X core^19^. These anti-correlations of the δ^18^O records in the ostracode shells of Lake Nakaumi could be ascribed to the differences in water mass that affected the core sites. Complicated water discharge patterns are currently observed in Lake Nakaumi: A large volume of low-salinity water that outflows from the surface of the lake causes an increase in the inflow of seawater at the bottom of the lake from the Sea of Japan^21,22^. The location of the X core is far from the mouth of the Iinashi River, which transports a large volume of freshwater that is discharged into the lake, which does not occur at the site of the N2015 core (Fig. 1). In addition, the X core is located in the area where seawater directly flows from the entrance to the central part of the lake at the bottom. Thus, it is estimated that marine water strongly affected the bottom portion of the X core during periods of heavy precipitation. The positive correlations between the δ^18^O records of stalagmites in Wanxiang Cave and the δ^18^O records of adult ostracode shells from the X core are identical since AD 1100, which is approximately equivalent to the timing of the environmental shift to closed blackish lake conditions (Fig. 2). Furthermore, the common peaks of the three records in Lake Nakaumi and Wanxiang Cave support that the δ^18^O records of adult ostracode shells in the lake represent the EASM variations. Hence, these observations infer that the centennial-scale δ^18^O records of adult ostracode shells in the lake reflect the intensity of the EASM; however the δ^18^O records of ostracode shells taken from the X core show anti-variations before AD 1100. Yamada *et al*.^19^ interpreted these anti-variations between the δ^18^O records of ostracode shells taken from the X core and the δ^18^O records of stalagmites in Wanxiang Cave as the northwestern migration of the monsoon limit. However, additional δ^18^O records from ostracode shells in the present study reveal that the anti-correlation was probably caused by the local water circulation conditions in the lake.

### EASM characteristics over the last 3 kyrs

To remove the δ^18^O variations affected by the regional environmental changes and identify the centennial-scale EASM variations, standardized δ^18^O values of ostracode shells were calculated and compared to the δ^18^O values of stalagmites from Dongge Cave^4^, Heshang Cave^13^, and Wanxiang Cave^20^ in China and cosmogenic atmospheric ^14^C^23^. Furthermore, pollen-based reconstructed precipitation in Gonghai Lake, China^24^ was also compared to our data (Supplementary Fig. S1). The standardized values and their comparisons show the variability of the multi-centennial-scale EASM intensity and coherence in Asian monsoonal regions (Fig. 3). Some weak millennial-scale EASM events that existed in the records from Baeg-nyong Cave, the Korean Peninsula^25^, Niu Cave, Central China^26^ and Dongge Cave^8^ were identical. Some of the events were correlated with the weak period in our records, inferring that some of the millennial-scale EASM weak periods found in the present study coincide with the EASM variations of a much longer timescale.

**Figure 4:**
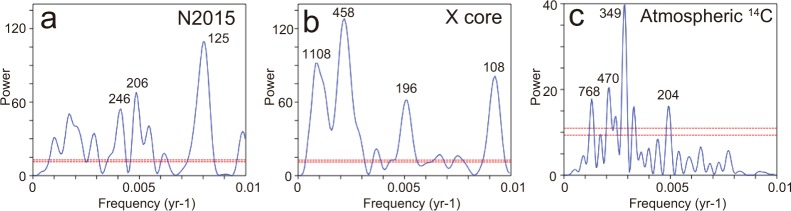
Spectral analysis of standardized values. (**a**) δ^18^O records in ostracode shells from the N2015 core. (**b**) δ^18^O records in ostracode shells from the X core^19^. (**c**) Atmospheric ^14^C^23^. Numbers show the periodicities for the main spectral peaks. The 0.01 and 0.05 significance levels are shown as dashed red lines.

**Figure 5:**
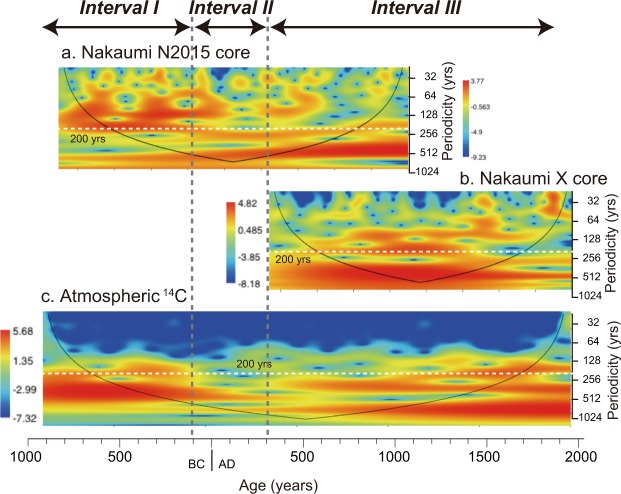
Wavelet spectra for the standardized values. (**a**) δ^18^O records in ostracode shells of *Bicornucythere bisanensis* from the N2015 core in Lake Nakaumi. (**b**) δ^18^O records in ostracode shells of *B*. *bisanensis* from the X core in Lake Nakaumi^19^. (**c**) Atmospheric ^14^C^23^. The power is represented by colours ranging from deep blue (weak) to red (strong). A ~200 yr cycle is shown by a white dotted line. The thin black lines indicate the 95% significance level.

A common ~200 yr periodicity of both the core and atmospheric ^14^C was identified from the spectral analysis of the standardized values of the δ^18^O records of adult ostracode shells (Fig. 4). Furthermore, wavelet analysis inferred that the ~200 yr periodicity dominated between BC 900 and BC 100 in both the N2015 core and atmospheric ^14^C. No distinguishable cycle was recognized between BC 100 and AD 400. Strong 300–500 yr periodicities, which differ from the cycles found in atmospheric ^14^C, were observed since AD 400 in the N2015 and X cores (Fig. 5). In addition to these periodicities, the comparisons of the EASM records infer that the EASM variations over the last 3 kyrs were divided into three intervals according to the timing of EASM intensity and relations to solar activity (Fig. 3). Interval I was recognized between BC 800 and BC 100. Strong EASM intervals in Lake Nakaumi were precisely correlated with those in Dongge Cave^4,8^ and approximately correlated with those in Heshang Cave^13^, in addition to the predominance of the 200 yr periodicity. Interval II, which occurred between BC 100 and AD 400, was characterized by EASM variations with relatively small amplitudes from Lake Nakaumi, as well as small amplitudes in the atmospheric ^14^C variations. However, distinguished small amplitudes were never exhibited in the stalagmite records from East Asia. There was no correlation between the EASM intensity and periodicity in both our records and those from stalagmites. The common 300–400 yr periodicities that were never recognized from atmospheric ^14^C were dominant in both cores from Lake Nakaumi during Interval III (AD 400–1800). The EASM intensity in Lake Nakaumi agrees with those in Wanxiang Cave, which is located farthest north of the three caves, whereas the EASM intensity from Lake Nakaumi disagrees with the stalagmite records from Dongge and Heshang Caves during this interval.

### Main factors of EASM variations and their shifts

The 200 yr cycles found in Interval I which are called the De Vries/Suess cycle, are frequently identified in climate change records such as temperature and precipitation variations during the Holocene^12,27–31^. These cycles are thought to be associated with solar activity because 200 yr periodicity has been observed in the production rates of the cosmic isotopes ^10^Be and ^14^C^32,33^. Several previous investigations identified correlations between the EASM intensity and solar cycles, particularly in millennial and orbital time scales^5,34,35^. The cycle with a periodicity of 200 yr was found in the EASM intensity derived from the δ^18^O records in stalagmites from Dongge Cave, southern China, as well as 80 and 340 yr cycles during the last 4.2 kyrs^8^. Coherent centennial-scale EASM variations within the regions affected by the EASM and the predominance of the 200 yr periodicity between BC 800 and BC 100 imply that solar activity strongly affected the EASM intensity, and the EASM variability was controlled by the common occurrence of the system in East Asia.

Conversely, the EASM variations and the factors controlling them are slightly inconsistent since BC 100. Limited association of the EASM variations with solar activity was confirmed. The EASM variations in the Japanese archipelago, expanding to Wanxiang Cave, central China, differed from those covering Dongge Cave, southern China, suggesting that consistent EASM variations were restricted to a particular region after BC 100. The precipitation-based ENSO cycle indicated by the high intensity of Launa Pallcacocha sediments, southern Ecuador^36^, coincided with other broad climatic records from East Asia^26,37,38^, suggesting that this record is one of the representative ENSO variation during the late Holocene. The enhanced ENSO (prominent El Niño) represents a good correlation to the strong centennial-scale EASM variations from Lake Nakaumi after AD 400 (Fig. 3). This observation reveals that EASM fluctuations are linked to sea surface temperature anomalies in the equatorial Pacific Ocean related to ENSO since BC100. A strong correlation between the centennial-scale EASM intensity and ENSO was observed from stalagmite records over the past 3 kyrs^26^ as well as for the Asian summer monsoons derived from other proxy records^39–41^. Lu *et al*.^42^ suggested that ENSO was responsible for drought and flooding in China during the late Holocene, although these relations were never found in the mid-Holocene. Generally the weak millennial-scaled EASM events were correlated with the prominent ENSO periods^26,39,41^, which is opposite of the correlations found in this study. Enhanced ENSO causes low surface temperatures in the western equatorial Pacific Ocean. This situation induces the development of relatively cold air in the middle latitudes of the western Pacific Ocean. As a result, high-pressure conditions occur in the mid-latitudes of the Pacific Ocean, which produces a low EASM intensity^43–45^. However, recent investigations identified regional contrast in precipitation between low- and mid-latitudinal areas in the Northern Hemisphere of the western Pacific during the past millennia. These regional variations are induced by Pacific Walker circulation variations^46^ and meridional circulation^47^ which profoundly affect precipitation in tropical Pacific regions and the mean position of the intertropical convergence zone. Hence our finding of a positive correlation between EASM intensity and ENSO is explained by these phenomena, although the mechanisms of EASM variation are complicated.

Unless ENSO showed a prominent coincidence with the EASM variations in and around the Japanese archipelago, solar activity^8,9,48^ and North Atlantic circulation^9^ were proposed as factors that affected the centennial-scale EASM variations in southern China, as well as air temperature in North China^47^ during the last 2 kyrs. In addition, the stalagmite δ^18^O records from Baeg-nyong Cave in Korea indicated that the EASM variations accompanying the periodicity that was shorter than the millennial scale occurred independently from the North Atlantic climate system over the past 4 kyrs^25^, corresponding to our results. This evidence infers that the prominent factors that affected the centennial-scale EASM variations varied in each region since 2 kyr, inferring that the EASM system around the Japanese archipelago was separated at least from southern and northern China.

Hence, we observed that solar activity was the most exclusive factor that controlled the EASM prior to BC 100, and then, the main factor modulating the EASM variations changed, at least in and around the Japanese archipelago. Furthermore, the primary factor was not common throughout the EASM region, although ENSO-induced sea surface temperature gradients were the main factor in and around the Japanese archipelago since BC 100. A prominent shift in climate variations was found at approximately 2 kyr, which is termed the “2 kyr shift”^5^. The relationships between the millennial-scale EASM intensity from the δ^18^O records of stalagmites in Sanbao Cave and insolation shifted from positive to negative at 2 kyr. The 2 kyr shift agrees with our findings that the EASM system changed drastically at BC 100. A few reasons for the 2 kyr shift have been proposed. The shift was invoked by the strengthening of the Atlantic meridional overturning circulation^5^ and increases in greenhouse gasses^42^, although there is little evidence for this phenomenon. Monsoon variations are affected by external forcing, including solar insolation and internal feedback, such as Atlantic meridional overturning circulation, high latitude climate changes and ENSO. As previously mentioned, solar insolation is an important factor that directly controls the EASM. Insolation at 30°N in July has decreased from BC 800 to present^49^ (Fig. 3a), although the amplitude of this decrease is small. Earth’s climate as well as the Indian summer monsoon are quite sensitive to small variations in solar irradiance^50^. The reduction in insolation might have induced a shift in the factors modulating the centennial-scale variations in the EASM intensity from solar activity to external factors at BC 100 in both the Japanese archipelago and Sanbao Cave. Subsequently, indirect relationships between EASM variations and solar insolation were recognized. The amplitude of the orbital-scale EASM variation is not necessarily consistent with that of insolation during the periods in which insolation was weak, particularly during glacial periods of 20 kyr, 70 kyr and 110 kyr BP, suggesting that there were nonlinear responses for the centennial-scale EASM variations that occurred during those intervals^5^.

Our results and compiled data propose that insolation variation was a primary factor of the centennial-scale EASM variations in all regions affected by the EASM during periods of high insolation, as noted in previous studies. However, we suggest that the factor exerting the EASM variations, at least at the centennial scale, has varied in the past due to low solar insolation intensity and was variable throughout the monsoonal areas in some situations. From this point of view, similar turnovers of the dominant factors for the EASM variations were observed in Gonghan Lake in China^24^, Qinghai Lake, northeastern Tibetan Plateau^51^, and Lake Biwa, central Japan^52^. In particular, the facts that solar activities are partly responsible for the variations in the Asian summer monsoon and the dominant factor changed after 32 ka in Qinghai Lake support our idea that the dominant forcing of EASM variations most likely changed according to the intensity of solar insolation.

## Methods

### N2015 core

The N2015 core (35°28.205′N, 133°10.894′E) was taken from the central part of Lake Nakaumi at a depth of 6.3 m in 2015 by an air-pressured piston corer. The core was mostly composed of mud bearing shell fragments. The core sediments were cut into one-cm-thick slices and washed on a 63 µm opening sieve. Then, the residual materials were dried at room temperature. Eight samples of plant materials and molluscan shells were used for ^14^C dating (Supplementary Table S2). The age model was calculated by a cubic equation based on the median age of eight calculated ^14^C ages, suggesting that the age of the core dated back to BC 800 and deposition had been successive (Supplementary Fig. S2). An average marine reservoir age of 400 yrs was subtracted from each ^14^C age following the methods of Yamada *et al*.^19^ and Ota *et al*.^53^.

### Isotope analysis

More than four individuals of complete adult shells of *B*. *bisanensis*, which is currently an abundant species in the closed bay and brackish lake bottoms around the Japanese Island, were picked from each residual sediment sample (Supplementary Table S1) and cleaned according to the procedures in Yamada *et al*.^19^. The δ^18^O and δ^13^C records of the ostracode shells from a total of 176 measurements were explored using an IsoPrime mass spectrometer with an automated carbonate preparation system (IsoPrime Multiprep) (Isoprime Ltd., U.K.) at the Center for Advanced Marine Core Research, Kochi University, Japan. The δ^18^O and δ^13^C values were calculated by means of six repeated measurements. The values are expressed on a VPDB (Vienna Pee Dee Belemnite) scale. The analytical precision was confirmed by repeated measurements of standard IAEA-CO-1 (δ^13^C: +2.49‰, δ^18^O: −2.4‰) and IAEA603 (δ^13^C: +2.46‰, δ^18^O: −2.37‰). The analytical errors of δ^13^C and δ^18^O were ±0.05‰ and ±0.06‰, respectively.

### Statistical analysis

Standardized δ^18^O values of ostracode shells, stalagmite and atmospheric ^14^C were calculated by subtracting the 500 yr running mean from the 50 yr running mean and dividing it by the standard deviation, following the methods in Wanner *et al*.^54^ and Yamada *et al*.^19^. Spectral and wavelet analyses were performed on the annual standardized δ^18^O values in ostracode shells from N2015, δ^18^O values in ostracode shells from the X cores, and atmospheric ^14^C, using PAST software^55^. The annual standardized δ^18^O values in ostracode shells were calculated by interpolating between the measurements from ostracode shells.

## Supplementary information

Supplementary information

## Data Availability

All data are included in this published article and its Supplementary Information files.
