# Supplementary material for: The variations in the East Asian summer monsoon over the past 3 kyrs and the controlling factors
Source: Sci Rep. 2019 Mar 22;9:5036. doi: 10.1038/s41598-019-41359-y (PMC6430807; doi:10.1038/s41598-019-41359-y)
Supplement: Supplementary file 1 — Supplementary information [file 41598_2019_41359_MOESM1_ESM.pdf]

Supplementary information

The variations in the East Asian summer monsoon over the past 3 kyrs and the controlling factors

Katsura Yamada, Kazuma Kohara, Minoru Ikehara and Koji Seto

Supplementary Table S1 Carbon and oxygen isotope values for the ostracode shells of *Bicornucythere bisanensis* in N2015 core taken from Lake Nakaumi, southwestern Japan.

Sample No.	Median core depth (cm)	Age (year)*	$\delta^{13}C$ VPDB (‰)	$\delta^{18}O$ VPDB (‰)
N2015-207	206.5	1073	-5.116	-0.094
N2015-208	207.5	1067	-5.844	-0.587
N2015-209	208.5	1061	-5.329	-1.027
N2015-210	209.5	1055	-5.553	-0.360
N2015-211	210.5	1048	-5.502	-0.880
N2015-212	211.5	1042	-5.615	-0.674
N2015-213	212.5	1036	-5.234	-0.698
N2015-214	213.5	1030	-5.095	-0.294
N2015-215	214.5	1023	-5.081	-0.736
N2015-216	215.5	1017	-5.876	-0.526
N2015-217	216.5	1011	-5.705	-0.856
N2015-218	217.5	1004	-5.180	-0.383
N2015-219	218.5	998	-5.355	-0.729
N2015-220	219.5	991	-5.379	-0.698
N2015-221	220.5	985	-5.170	-0.384
N2015-222	221.5	978	-5.202	-0.462
N2015-223	222.5	972	-5.127	-1.347
N2015-224	223.5	965	-5.751	-0.187
N2015-225	224.5	958	-5.426	-0.279
N2015-226	225.5	951	-5.468	-0.370
N2015-227	226.5	945	-5.251	-0.262
N2015-228	227.5	938	-4.424	-1.150
N2015-229	228.5	931	-5.089	-0.200
N2015-230	229.5	924	-5.288	-0.093
N2015-232	231.5	910	-5.711	-0.131
N2015-233	232.5	903	-5.744	0.349
N2015-234	233.5	896	-5.668	0.042
N2015-235	234.5	889	-5.501	0.265
N2015-236	235.5	882	-4.888	-0.407
N2015-237	236.5	874	-5.011	-0.234
N2015-238	237.5	867	-5.178	-0.141
N2015-239	238.5	860	-5.289	-0.021
N2015-240	239.5	853	-5.421	0.005
N2015-241	240.5	845	-5.520	0.002
N2015-242	241.5	838	-4.301	-0.336
N2015-243	242.5	830	-4.748	-0.204
N2015-244	243.5	823	-5.096	-0.158
N2015-245	244.5	815	-5.173	0.025
N2015-246	245.5	808	-5.918	-0.266
N2015-247	246.5	800	-5.336	-0.230
N2015-248	247.5	792	-4.648	-0.347
N2015-249	248.5	784	-4.831	-0.139
N2015-250	249.5	777	-5.756	-0.605
N2015-251	250.5	769	-5.533	-0.351
N2015-252	251.5	761	-5.769	-0.371
N2015-253	252.5	753	-5.819	-0.017
N2015-254	253.5	745	-5.107	-0.185
N2015-255	254.5	737	-4.789	-0.250
N2015-256	255.5	729	-5.260	-0.375
N2015-257	256.5	721	-5.192	-0.337
N2015-258	257.5	712	-5.029	-0.144
N2015-259	258.5	704	-5.744	-0.287
N2015-260	259.5	696	-4.930	-0.108
N2015-261	260.5	688	-5.536	-0.242
N2015-262	261.5	679	-5.774	-0.336
N2015-263	262.5	671	-5.321	-0.155
N2015-264	263.5	662	-5.120	-0.273
N2015-265	264.5	654	-4.256	-0.484
N2015-266	265.5	645	-5.354	-0.096
N2015-267	266.5	637	-5.055	-0.159
N2015-268	267.5	628	-5.365	-0.526
N2015-269	268.5	619	-4.847	-0.486
N2015-270	269.5	610	-5.410	0.031
N2015-271	270.5	602	-4.941	-0.344
N2015-272	271.5	593	-5.121	-0.485
N2015-273	272.5	584	-5.593	-0.145
N2015-274	273.5	575	-5.462	-0.259
N2015-275	274.5	566	-5.192	-0.285
N2015-276	275.5	557	-5.992	-0.584
N2015-277	276.5	548	-5.343	-1.050
N2015-278	277.5	538	-5.290	-0.064
N2015-279	278.5	529	-6.396	-0.223
N2015-280	279.5	520	-5.463	-0.271
N2015-281	280.5	510	-5.888	-0.061
N2015-282	281.5	501	-5.493	0.243
N2015-283	282.5	492	-5.604	-0.149
N2015-284	283.5	482	-5.607	-0.625
N2015-286	285.5	463	-5.986	-0.311
N2015-288	287.5	443	-5.203	0.180
N2015-289	288.5	434	-4.660	0.053
N2015-290	289.5	424	-4.608	-0.190
N2015-291	290.5	414	-4.803	0.121
N2015-292	291.5	404	-4.745	0.335

Sample No.	Median core depth (cm)	Age (year)*	$\delta^{13}C$ VPDB (‰)	$\delta^{18}O$ VPDB (‰)
N2015-293	292.5	394	-5.361	0.518
N2015-294	293.5	384	-5.022	-0.037
N2015-295	294.5	374	-5.104	0.367
N2015-296	295.5	364	-4.487	-0.058
N2015-297	296.5	353	-5.371	0.163
N2015-298	297.5	343	-5.339	-0.673
N2015-299	298.5	333	-4.789	0.018
N2015-300	299.5	322	-5.049	-0.029
N2015-301	300.5	312	-4.996	-0.482
N2015-302	301.5	301	-5.409	-0.200
N2015-303	302.5	291	-4.663	0.647
N2015-304	303.5	280	-4.804	-0.173
N2015-305	304.5	269	-4.827	-0.070
N2015-306	305.5	258	-4.645	-0.149
N2015-307	306.5	248	-5.232	-0.332
N2015-308	307.5	237	-5.531	-0.147
N2015-309	308.5	226	-5.056	-0.157
N2015-310	309.5	215	-5.388	-0.052
N2015-311	310.5	204	-4.483	-0.285
N2015-313	312.5	181	-5.549	0.197
N2015-314	313.5	170	-5.575	-0.269
N2015-315	314.5	159	-4.504	-0.454
N2015-316	315.5	147	-4.638	0.143
N2015-317	316.5	136	-5.057	0.042
N2015-318	317.5	125	-5.727	-0.342
N2015-319	318.5	113	-5.295	-0.051
N2015-320	319.5	101	-4.742	-0.044
N2015-321	320.5	90	-5.190	-0.176
N2015-324	323.5	54	-4.852	-0.282
N2015-327	326.5	18	-4.712	0.095
N2015-332	331.5	-43	-4.067	-1.031
N2015-333	332.5	-55	-4.553	0.396
N2015-334	333.5	-68	-4.701	-0.224
N2015-337	336.5	-106	-5.102	-0.007
N2015-338	337.5	-119	-5.045	0.369
N2015-339	338.5	-132	-4.547	-0.004
N2015-340	339.5	-144	-5.101	-0.351
N2015-341	340.5	-157	-4.782	-0.311
N2015-342	341.5	-170	-4.766	0.294
N2015-343	342.5	-184	-5.079	-0.029
N2015-344	343.5	-197	-5.143	0.282
N2015-345	344.5	-210	-5.702	-0.202
N2015-346	345.5	-223	-5.236	0.306
N2015-347	346.5	-237	-4.544	0.407
N2015-348	347.5	-250	-5.335	0.251
N2015-349	348.5	-264	-5.770	-0.634
N2015-350	349.5	-277	-4.542	0.184
N2015-351	350.5	-291	-5.273	-1.086
N2015-352	351.5	-305	-5.743	0.117
N2015-354	353.5	-332	-5.409	0.586
N2015-355	354.5	-346	-5.080	0.829
N2015-356	355.5	-360	-4.786	-0.171
N2015-357	356.5	-374	-4.296	0.333
N2015-358	357.5	-389	-4.562	0.183
N2015-359	358.5	-403	-4.647	-0.103
N2015-360	359.5	-417	-5.028	0.007
N2015-361	360.5	-432	-3.855	-0.453
N2015-362	361.5	-446	-5.736	0.318
N2015-363	362.5	-460	-4.761	-0.289
N2015-364	363.5	-475	-5.602	-0.067
N2015-365	364.5	-490	-5.154	0.317
N2015-366	365.5	-504	-4.484	0.207
N2015-367	366.5	-519	-5.285	0.164
N2015-368	367.5	-534	-5.043	0.083
N2015-369	368.5	-549	-4.545	0.706
N2015-370	369.5	-564	-4.424	-0.368
N2015-371	370.5	-579	-4.970	0.373
N2015-372	371.5	-594	-5.027	0.241
N2015-373	372.5	-610	-4.294	0.865
N2015-374	373.5	-625	-5.226	0.102
N2015-375	374.5	-640	-4.190	-0.653
N2015-376	375.5	-656	-4.273	-0.347
N2015-377	376.5	-671	-4.835	-0.124
N2015-378	377.5	-687	-4.358	-0.389
N2015-379	378.5	-703	-5.274	0.501
N2015-380	379.5	-718	-5.214	0.264
N2015-381	380.5	-734	-5.065	0.413
N2015-382	381.5	-750	-4.471	0.070
N2015-383	382.5	-766	-4.653	0.131
N2015-384	383.5	-782	-4.833	0.051
N2015-385	384.5	-798	-4.640	0.245
N2015-386	385.5	-815	-4.901	-0.254
N2015-387	386.5	-831	-4.619	0.216

*Negative values indicate year of BC.

Supplementary Table S2 ^{14}C age for two plant materials and six molluscan shells from N2015 core in Lake Nakaumi, western Japan.

Sample No.	Labo ID	Core depth (cm)	Materials	Measured radiocarbon age (y BP)	$\delta^{13}C$ (‰)	Conventional radiocarbon age (y BP)	Calendar age (2σ) (year)	Median calendar age (year)
N2015-5	420839	5	Mollusca (*Ruditapes philippinarum*)	80±30	-0.1	490±30	Cal AD 1715 to Post 1950	AD 1832.5
N2015-69	420840	69	Mollusca (*Paphia undulata*)	250±30	-0.6	650±30	Cal AD 1625 to 1695	AD 1660
N2015-102	420841	102	Mollusca (unknown)	330±30	+1.1	760±30	Cal AD 1485 to 1635	AD 1560
N2015-230	420843	230	Mollusca (*Paphia undulata*)	1100±30	+0.5	1520±30	Cal AD 790 to 955	AD 872.5
N2015-277	420844	277	Plant material	1590±30	-28.0	1540±30	Cal AD 425 to 595	AD 510
N2015-292	420845	292	Mollusca (unknown)	1540±30	0.0	1950±30	Cal AD 390 to 535	AD 462.5
N2015-371	420846	371	Mollusca (*Paphia undulata*)	2340±30	+0.8	2760±30	Cal BC 660 to 405	BC 532.5
N2015-384	420847	384	Plant material	2760±30	-30.2	2670±30	Cal BC 890 to 875 and Cal BC 845 to 800	BC 845

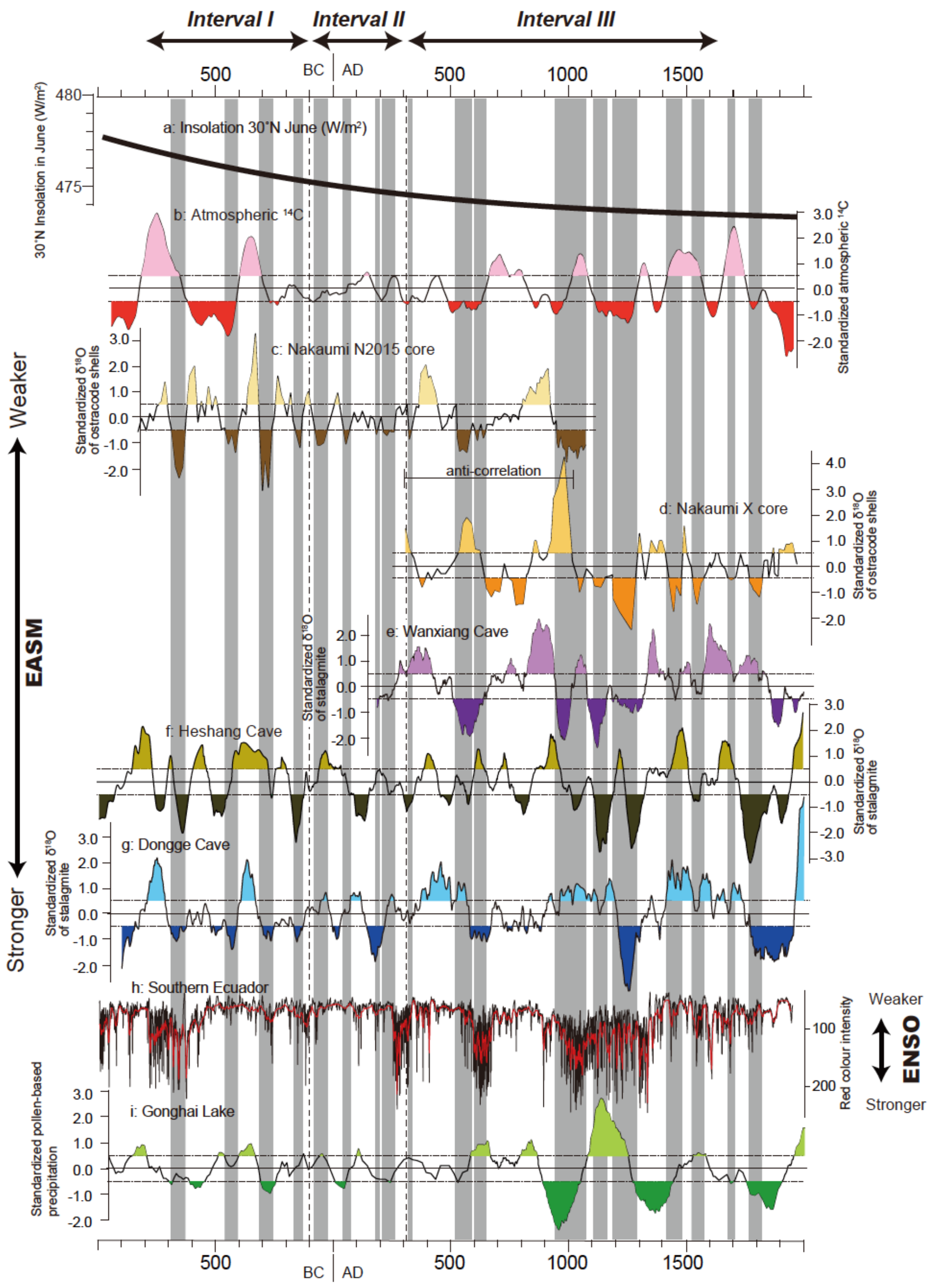

Supplementary Figure S1 Comparisons of EASM records in the monsoonal region and climatic records. Please refer the captions in Figure 3 for a–h. i, Standardized pollen-based precipitation from Donghai Lake, central China[24]. No common trend and similar periodicities between the pollen-based precipitation and our $\delta^{18}O$ records from Lake Nakaumi were

observed. The inconsistency infers complexity of monsoon variations through the EASM regions and may represents regional variation of EASM.

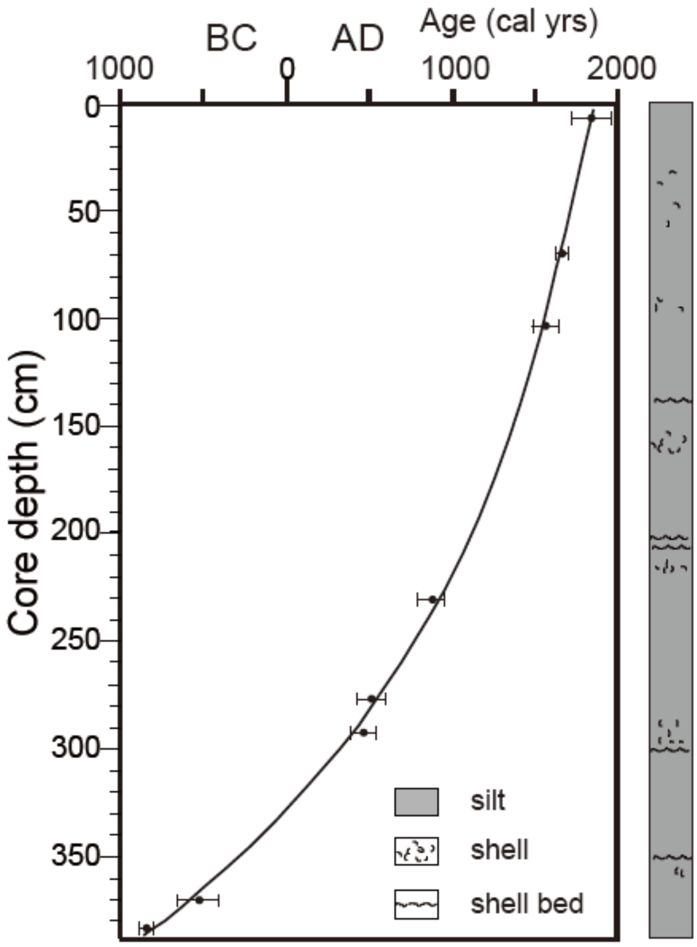

Supplementary Figure S2 Age model of the N2015 core in Lake Nakaumi, southwestern Japan.
